# Medical students during the COVID-19 pandemic: Considerations and arguments on their clinical engagement

**DOI:** 10.30476/JAMP.2021.89092.1363

**Published:** 2021-10

**Authors:** PANAGIOTIS STACHTEAS, NIKOLAOS VLACHOPOULOS, EMMANOUIL SMYRNAKIS

**Affiliations:** 1 Laboratory of Primary Health Care, General Practice and Health Services Research, Faculty of Medicine, Aristotle University of Thessaloniki, Greece

**Keywords:** COVID-19, Pandemics, Medical education, Medical students, Role

## Abstract

During the recent COVID-19 pandemic, the clinical exposure of medical students has been hindered while the impact on medical education is under investigation. The potential negative impact of medical students on transmission rates, along with the shortages of Personal Protective Equipment (PPE), their lack of proper training, and their limited ability to work independently, give rise to reasonable concerns regarding their involvement in pandemic management. Nevertheless, the heavy workload could soon provoke severe deficiencies in the frontline medical workforce. Hence, the possibility of covering gaps in human resources by effectively deploying students should not be rejected in advance. Additionally, a pandemic poses a valuable learning opportunity for high-quality medical education.

The aim of this commentary is to present a discussion with supporters and opponents of medical student engagement in the pandemic management and their involvement in placements requiring physical contact with patients. We also attempt to elucidate the necessary conditions for the students’ possible involvement in clinical settings.

## Introduction

The novel Coronavirus SARS-COV-2, responsible for the COVID-19 disease, emerged from Wuhan, the capital city of the Hubei province in China, but rapidly spread geographically, affecting millions of people worldwide. This health crisis poses an unprecedented challenge for medical education. Since the pandemic outbreak, the disruption of medical training was inevitable due to the implementation of social distancing, the universal hindrance of clinical exposure for many medical students, and the halt of clinical attachments and electives to curtail transmission ( 1 - 3 ). 

In this COVID-19 pandemic, medical educators across the globe have been forced to immediately generate distance learning environments by converting in a few weeks the majority of face-to-face theoretical sessions to online sessions ( 4 - 6 ). Although current evolving web-based ventures can host - and therefore substitute - the majority of didactic lectures of undergraduate curriculum, student clinical exposure remains an indispensable part of medical education, particularly affecting the transition from student to doctor ( 7 , 8 ). Nevertheless, general dissidence and uncertainty has led to varying attitudes among institutions regarding student involvement in clinical settings. Thus, it is more than reasonable for education providers to wonder whether senior medical students should continue their training in clinical rotations and if so, which are the appropriate circumstances under which medical students could be exposed to suspected patients.

### The argument against senior medical student involvement

To begin with, many medical schools have canceled activities with patient contact to assure student safety and to decrease the number of possible vectors. In general terms, students and residents are not always properly trained to follow hygiene protocols, especially in conditions of increased pressure ( 9 - 10 ). Compared to older doctors who are more likely to develop symptoms, students are more likely to be asymptomatic carriers, and, as a result, they may more easily acquire the virus during training and they could potentially spread it to unaffected patients without even knowing ( 2 , 3 , 5 , 11 ). By decreasing overcrowding caused by non-essential personnel (including medical students) in healthcare settings, medical schools will contribute to national and global efforts to "flatten the curve" (decrease transmission rates) ( 1 , 6 - 7 ).

Secondly, students’ involvement with suspected or confirmed cases calls for additional Personal Protective Equipment (PPE) (gloves, masks, respirators, goggles, face shields, and gowns), shortages of which have already been noted worldwide ( 12 - 14 ). In terms of effective allocation of PPE to essential personnel, especially in countries with significant deficiencies, canceling clinical residencies seems quite reasonable ( 1 , 7 , 9 , 11 ). 

From another viewpoint, students demand more attention and resources than the benefit they provide; they are short on experience and ability to work independently ( 2 ). In canceling classes and clinical rotations, medical schools hope not only to promote social distancing but also to minimize the teaching burden on the frontline health workforce, which is already assigned with the demanding task of offering hospitalization to infected patients ( 3 , 7 ). Shouldering simultaneously the extra weight of student education and supervision seems impractical, so prioritizing patient care, safety, and pandemic control over student learning seems justified ( 1 , 9 ). 

Furthermore, the uncertain clinical environment may affect medical staff’s mental well-being, leading to frustration, distress, anxiety, and other adverse psychological outcomes of unpredicted magnitude, especially when they provide care beyond their competency ( 4 , 8 , 15 ). Finally, it can be argued that the primary goal of medical students is to receive a proper and thorough education to graduate, and that they are in no case officially paid employees who are covered by policies and obliged to offer their services to the healthcare system; further, medical students are learners and not essential workers in the COVID-19 pandemic management ( 11 ). 

### The argument in favor of senior medical student involvement

On the other hand, it is undeniable that the needs of the health workforce are augmented in such periods. The increasing number of cases, the recommendation for a 2-week self-isolation period for anyone who has tested positive for the virus, and the continuous amount of considerable clinical work unrelated to COVID-19 could soon limit the pool of available healthcare providers ( 1 , 2 , 7 ). In this framework, students could be embedded in the clinical environment in a capacity to cover the significant gaps in human resources left by doctors who are suffering from COVID-19 ( 5 , 6 , 8 ). Additionally, apart from their clear role as trainees, medical students are routinely involved in the efficient operation of healthcare structures by participating in many of the day-to-day procedures, such as history taking, patient examination, and decision-making through differential diagnostic thinking ( 3 , 14 ). 

To further enhance this point of view, student commitment to contribute is remarkable, with medical student organizations, even at the initial stages of this health crisis, willing to offer their services. Their deep conviction is that they have already developed a set of clinical skills and a solid knowledge background, which render them more useful than any other group of volunteers that could be drafted to support the overworked healthcare professionals. An online survey, which included over two hundred students from central Europe, revealed that 70% of the students signed up for voluntary commitment, while almost half of the unwilling students were either already employed in hospitals or unable to sign up due to underlying health problems ( 16 ). 

According to another perspective, the aging of the healthcare workforce further complicates the functional state of health systems. Considering the higher likelihood of comorbidities, and the increased morbidity and mortality of COVID-19 in the higher age range, the suggestion to involve retired, thus even older, doctors seems a quite dangerous practice ( 2 ). Students are less vulnerable to negative health outcomes from infection and less susceptible to COVID-19 complications, thus, they are a more appropriate workforce to lessen the healthcare systems’ extra burden than retired clinician volunteers ( 3 , 7 ). 

From a pedagogic point of view, student engagement in crisis management is a rare educational experience and a unique opportunity to realize the dynamic nature of medical knowledge ( 17 , 18 ). Medical students are the future medical workforce and this experience can critically contribute to the maturity of their professional identity through the development of their interprofessional collaborative skills (altruism, teamwork, sense of duty, courage) and through their improved adaptability, persistence, and competency ( 19 , 20 ). The 'real-life' learning may be invaluable, as there is a possibility of an upcoming peak in COVID-19 cases, when these students will be on the wards as residents. Medical educators must ensure that the newly-graduated workforce will be sufficiently trained and ready to deliver essential patient care, even in crises, highlighting the need for ’pandemic preparedness’ to be embedded in the medical curriculum ( 14 , 17 , 21 ). Besides, according to a recently published systematic review, all interventions ranging from simple classroom based interactive discussions to complex multimodal simulative experiences resulted in improved knowledge, skills, and attitudes towards participation in disaster medicine ( 22 ). Given that the longer-term response to COVID-19 is unknown, removing students from clinical placements may negatively affect their future preparedness and more generally, future workforce planning ( 1 ). 

### Principles for student involvement

The appropriate role of medical students in clinical programs during a pandemic is an issue of controversy amongst medical educators. In this direction, it would be rather efficient to form generally-accepted guidelines, which will clearly outline in which vacancies and under what scenarios students could be usefully deployed in clinical placements ( 2 , 14 , 20 ). Medical Deans Australia and New Zealand Inc (MDANZ) and the Australian Health Practitioner Regulation Agency (Ahpra), as well as the Association of American Medical Colleges (AAMC) and the British General Medical Council (GMC) have endeavored to provide a framework for the recruitment of medical students into such roles ( 2 , 6 , 11 , 14 ). Medical educators across the globe express related viewpoints, which will be analyzed further. 

Although medical students generally accept a social contract that includes possible hazardous duty, it is accepted that their involvement in health crises should be prioritizing safety with adequate access to PPE and knowledge of evidence-based up-to-date practices ( 6 , 9 , 14 , 20 ). To maximize their efficacy and guarantee their safety, emphasis should be placed on targeted and intensive training before their involvement, especially on handwashing, N95 / P2 mask fitting, and donning/doffing of protective clothing ( 2 ). Furthermore, in the event of severe illness because of inpatient activities, medical students should be covered by policies like comprehensive health insurance ( 11 ). 

In addition, proper and continuous supervision of students is considered essential to ensure the safety of both themselves and patients ( 2 , 3 , 5 , 14 ). This guidance can be achieved, without burdening healthcare systems, by recruiting retired doctors or other physicians who belong to vulnerable groups for severe infection who cannot contribute to the first response line. Quarantined doctors can also contribute remotely to distance education. Training/Supervision in COVID19-related structures can take place either by employees in these structures or in the form of peer education ( 5 ). Moreover, medical students who start working in new roles to tackle the pandemic outbreak should work within their competence level, based on their education and experience; in no case should it be expected to “step up” and act outside of their competence level ( 2 , 12 ). Last, but not least, student contribution and involvement in clinical placements during the current pandemic should be voluntary and by no means mandatory ( 3 , 17 ). 

### Suggested pathways

It is widely perceived that the student role in response to these unprecedented circumstances is far from clear. However, medical training cannot be halted in every threat of a pandemic, and the medical education community is obliged to organize coordinated responses. Nevertheless, the idea of a single holistic solution for all cases seems rather utopian. A more reasonable alternative would be the adoption of practices related to the prevailing conditions of each country. 

Naturally, this individualized decision for student involvement should depend on specific criteria. It is of paramount importance that the advantages of such a decision should outweigh the risks so that the cost-benefit ratio will be truly beneficial not only for the overall crisis management, but also for medical students' education and professionalism. This condition gains even bigger value in the crucial point when medical demand outpaces medical capacity ( 7 ). Indeed, if COVID-19 overwhelms the healthcare system’s capacity to provide excellent patient care, students should have the opportunity to massively contribute to this fight. But even when medical capacity is adequate, their desire to participate combined with the invaluable educational opportunity makes their clinical involvement a reasonable solution as long as safety protocols are observed.

## Strengths and limitations

In this article, we endeavored to describe in detail some of the lessons learned from medical educators during the current COVID-19 pandemic. To our knowledge, studies referring to the active role of medical students during pandemics and the necessary conditions for their participation in clinical and outpatient settings are heterogenous. We combined some of these muddled data in an attempt to multilaterally address this topic. Nevertheless, we are aware of some limitations of note. While we mainly focused on clinical settings and senior medical students, further research should look into the generalizability of these practices into non-clinical settings. In addition, a significant gap of sufficient research articles in the current relative bibliography was observed, the majority of which are based on descriptions of the experience gained by educators around the world. Thus, there is a need for longer-term studies to examine in detail the effects of the pandemic on medical education and the possible active contribution of students to its tackling, in order to draw safe conclusions.

## Conclusion

The outbreak of a large-scale contagious, infectious disease hampers inpatient education. Nevertheless, a pandemic poses a great learning opportunity for students in tackling a health crisis. Concurrently, student contribution seems to be precious for an overburdened healthcare system. Therefore, the option to effectively deploy students, mainly in outpatient positions and primary care settings, but also in direct patient activities, should be promoted under specific conditions and cannot be rejected in advance. The formation of a clear framework for students’ roles in such circumstances enhances the preparedness of the medical education community for related future challenges. 
